# Antimicrobial Effects of Four Intracanal Medicaments on *Enterococcus Faecalis*: An in Vitro Study

**Published:** 2014-07-05

**Authors:** Mohammad Ali Mozayeni, Ali Haeri, Omid Dianat, Ali Reza Jafari

**Affiliations:** a Department of Endodontics, Dental School, Iranian Center for Endodontic Research, Research Institute for Dental Sciences, Shahid Beheshti University of Medical Sciences, Tehran, Iran; b Private Practice, Tehran, Iran; c Dental Student, Students’ Research Committee, Shahid Beheshti University of Medical Sciences, Tehran, Iran

**Keywords:** Antibiotics, Anti-Infective Agents, Antimicrobial Agent, Calcium Hydroxide, Chlorhexidine, Canal Disinfectants, *Enterococcus Faecalis*

## Abstract

**Introduction**: The aim of this *in vitro* study was to evaluate the antimicrobial activity of four intracanal medicaments on *Enterococcus Faecalis (E. Faecalis).* **Methods and Materials:** Fifty extracted single-rooted human teeth were prepared with standard method. After contaminating the canals with *E. Faecalis*, the samples were divided into one control and four experimental groups (*n*=10). The teeth in each group were treated with one of the experimental medicaments, including calcium hydroxide (CH), 2% chlorhexidine gel (CHX), triple antibiotic paste (TAP) and nanosilver (NS). In control group, canals were filled with a neutral gel. Microbial samples were obtained from the roots after 7 days and optical density of the cultures was determined after 24 h of incubation. Optical density values were analyzed with one-way analysis of variance and Tukey’s post hoc tests. **Results:** CHX gel and TAP were significantly more effective against *E. Faecalis* than CH, which was also significantly more efficient than NS and normal saline. In the paper cone samples, CHX gel was more effective than TAP; however, samples obtained with sizes 2 and 4 Gate Glidden drills, indicated that TAP was much more efficient than CHX. Normal saline and NS had similar effects on *E. Faecalis*. **Conclusion:** NS gel was not efficient enough against *E. Faecalis*; however, TAP and CHX gel showed better antibacterial efficacy than CH and can be used as an alternative intracanal medicaments in root canal therapies.

## Introduction

Complete debridement and reduction of the bacterial infection from the root canal space seems to be necessary for long-term success of endodontic treatment [1, 2]. Endodontic infection is considered as a polymicrobial disease [2]. The eradication of microorganisms from the infected root canal system is a complicated task including instrumentation, irrigation and application of intracanal medicaments. It is noticeable that many researchers have shown that significant portions of the root canal walls remain untouched after mechanical instrumentation. Consequently, chemical irrigators and intracanal medicaments seem necessary for eradication of infected tissues and microorganisms in addition to mechanical debridement [3]. Persistence of microorganisms in apical third of the root canals leads to failure of treatments. 

*Enterococcus Faecalis* (*E. Faecalis)* has been found in 38% of the failed root canal-treated teeth [4]. The ability to tolerate the rough environmental changes which is believed to be due to its high alkali tolerance [5] and tubular invasion ability of this cocci which protects it from intracanal endodontic medicaments, has made *E. Faecalis* a treatment-resistant microorganism [6]. 

Calcium hydroxide (CH) is widely used as an intracanal medicament in endodontic therapy. The high pH of CH destroys the bacterial cell membrane and protein structures [7]. However, some evidences have shown that CH was inefficient against *E. Faecalis* and *Streptococcus Sanguinis* as they remained viable in the dentinal tubules [3, 6].

Another alternative root canal medication is chlorhexidine (CHX) gluconate with well-known broad-spectrum antimicrobial effects [7]. CHX molecule consists of two symmetric 4-cholorophenyl rings and two biguanide groups connected by a central hexamethylene chain. Due to the positively charged molecule, CHX interacts with negatively charged phosphate groups on the microbial cell wall and causes its leakage. Mechanism of action for CHX is its absorption onto the cell wall which causes cellular leakage and allows the CHX molecule to penetrate into the bacteria [7-9]. On the other hand it has been shown that the additional use of 0.2% CHX solution for canal irrigation can reduce the success of root canal treatment [7]. CHX has been shown to be more effective in eliminating microorganisms like *E. Faecalis *which resisted against CH inside the dentinal tubules [9-11]. Also sequential use of CHX and sodium hypochlorite (NaOCl) solutions for root canal irrigation had a negative impact on treatment, probably due to the effects of their interaction products [7].

The wide antimicrobial effect of silver is well-known and it has been used in different fields in medicine such as bandages, wound dressings, ointments and surfaces of catheters, implants and prostheses [12, 13]. Even low concentrations of silver can inhibit bacterial growth. Recently various antibacterial materials containing silver have been generated [14]. Nanoparticles are clusters of particles in the size range of 1-100 nm. Silver nanoparticle, (Nanosilver; NS) probably has the ability to inhibit the bacterial growth due to their larger surface area, which is an important factor in antimicrobial activity [14]. NS has well-known antimicrobial effect and is widely used in different areas, such as clothes, catheters, electric home appliances, and biomedical implants [15]. Antimicrobial properties of NS exert via interaction with the sulfur containing proteins present in the bacterial cell membrane and also with phosphorus containing DNA [16].

Recently a mixture of metronidazole, ciprofloxacin and minocycline, also known as the triple antibiotic paste (TAP), has been used as an intracanal medicament for disinfecting the root canal during tissue regeneration [17]. Metronidazole is a wide spectrum bactericidal antibiotic [18]. *In vitro* experiments have shown that 10 µg/ml metronidazole can eliminate more than 99% of bacteria found in infected root canals. On the other hand, increasing the concentration of metronidazole could not kill all the bacteria. Therefore to sterilize the infected root canal, we need other antibiotics such as ciprofloxacin and minocycline [18]. Some researchers have reported that the TAP can sterilize root dentin. Also Adl *et al.* [17] showed that TAP can effectively eliminate *E. Faecalis*. For this purpose, this investigation compared the *in vitro* antimicrobial activity of four gel form intracanal medicaments (CH, 2% CHX, TAP and NS) against *E. Faecalis*.

## Methods and Materials

Fifty extracted, straight single-rooted human teeth were collected. Teeth with curved roots, canal calcifications and root caries were excluded from the study. Bone, calculus and soft tissues on the root surfaces were slightly removed by means of a periodontal curette. Collected teeth were placed in 5.25% NaOCl for 1 h in order to disinfect the root surfaces and the samples were stored in 0.9% physiological saline. The crowns were cut perpendicular to the long axis of the teeth from cementoenamel junction (CEJ) with a diamond disc in conjunction with physiological saline irrigation and the root lengths were cut and standardized to 16 mm.

Canals were evaluated for apical patency and checked to have one canal with similar size to a #15 K-file (Mani, Tochigi, Japan). The root canals were instrumented 0.5 mm beyond the apex using the ProTaper rotary system (Dentsply Maillefer, Ballaigues, Switzerland) with 5.25% NaOCl irrigation. After root canal preparation to size F3, the samples were placed in a 4-min ultrasonic bath with 17% ethylenediaminetetraacetic acid (EDTA) for smear layer removal. In order to complement the effects of EDTA, a flush with 5.25% NaOCl for 5 min was done [19]. Finally aiming at removing the remnants of EDTA and NaOCl, each tooth was rinsed with 10 mL of physiological saline. Each root specimen was placed in a microtube containing 2 mL of tryptic soy broth (TSB, Difco Laboratories, Detroit, Mich., USA) and then autoclaved twice for 30 min at temperature of 121° C and pressure of 15 PSI. After that, samples were stored in an incubator at 37° C for 24 h. Sterile TSB was removed by using sterile micropipettes from microtubes and then it was replaced by 1 mL of *E. Faecalis* suspension with standard concentration of 0.5 McFarland (1.5×10^8^ CFU/ml). The tubes were closed and incubated at 37° C for 1 day. Bacterial viability and purity were checked in 3 randomly-picked tubes. After that, samples were incubated for 21 days at 37° C. During incubation period, in order to prevent dehydration of the samples, TSB culture was changed every 3 days.

In order to prepare gel form of medicaments, methyl cellulose was used as an inert carrier. One g of methyl cellulose gel with concentration of 50 ppm and particle size of 20 nm, was separately added to 1 g of CH (Merck, Darmstadt, Germany), 1 g of 4% CHX solution, 1 g of TAP and 1 g of NS (NanoSilver Solution, Lotus Nanochemistry, Pasargad, Tehran, Iran). Also 1 g of methyl cellulose was added to physiological normal saline as a negative control group. In order to gain a gelatinous form, 3 drops of viscous polyethylene glycol 400 (PEG, Dae Jang, Gyeonggi-do, Korea) were added to each medicament.

Each prepared medicament was injected into 10 infected roots from coronal to apical. Ten teeth were chosen for the control group. Samples were returned to incubator at 37^°^ C. After 7 days of incubation, intracanal medicaments were removed from canals by irrigation with 10 mL of normal saline. Bacterial sampling was performed using a sterile #30 paper point. The paper point was left in the root canal for 1 min and then transferred to a solid Bile Esculin Agar (BBE) culture plate. After that, apical third of roots were drilled by #2 and 4 Gates Glidden drills (Dentsply, Maillefer, Ballaigues, Switzerland) to the depth of 200 and 400 µm into dentinal walls. 

**Figure 1:**
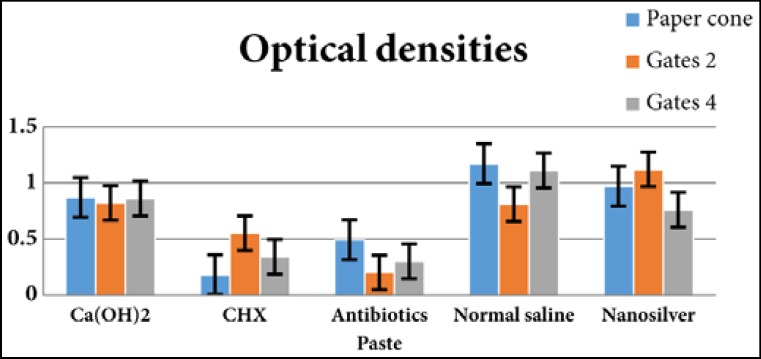
Optical density of different experimental groups obtained from root canal surface and dentinal depths of 200 and 400 µm

Dentin shaves were removed with Gates Glidden drills and a BHI (brain-heart infusion) broth (Difco Laboratories, Detroit, MI) saturated paper point. Prepared paper points were transferred to test tubes containing 2 mL of BHI aqueous culture. Turbidity test of tubes was measured by Spectrophotometer (Unico 2100, New Jersey, USA) after 24 h of incubation at 37^°^ C.

Optical density values among groups were analyzed with one-way analysis of variance and comparison between groups was done by Tukey’s post hoc test. *P* values less than 0.05 were considered as significant.

## Results

The optical densities of samples are presented in Figure 1. There was no significant differences in optical density of groups before treatment by intracanal medicaments (*P*=0.21). After 7 days, on the specimens of the paper cone, significant differences in optical densities were found between different intracanal medicaments (one-way analysis of variance: *P*=0.0001). In these samples, the activity of CHX and TAP, TAP, CH and NS, CH, NS and normal saline was within the same range. The most and the least antibacterial efficacy was noted in CHX and normal saline samples, respectively. On Gates Glidden #2 samples, there was a significant difference between optical densities of these specimens, as well. CHX and TAP, CHX, normal saline and CH, CH and NS showed the same range of antibacterial activity. In this group TAP and NS showed the most and the least antibacterial efficacy, respectively. In Gates 4 samples, a significant difference between optical densities were found (*P*<0.0001). The antimicrobial activity of TAP, CHX, NS and CH, NS, CH and normal saline was within the same range. TAP was the most effective medicament while normal saline was the least effective against *E. Faecalis*.

## Discussion

The golden rule of successful root canal therapy is infection elimination and three dimensional obturation of the canals to preclude subsequent reinfection [4]. However, current techniques of debridement leave parts of root canal space completely untouched by the instruments. *E. Faecalis *is associated with persistent apical periodontitis and resists elimination from root canals [7]. *E. Faecalis* has the capacity to proliferate in the deeper layers of dentine [20]. Thus the penetration of medicaments into dentinal tubules was evaluated by investigation of optical density of samples obtained by two different sizes of Gates Glidden drills (*i.e.* 2 and 4) in addition to paper cones. The results showed that the most effective medicament against *E. Faecalis* was TAP and after that in descending order were CHX, CH, NS and normal saline. Other studies also demonstrated that, 2% CHX gel was effective against *E. Faecalis* even 21 days after root dentine treatment [20]. In the study by Dametto *et al.* [21], 2% CHX gel and 2% CHX liquid significantly reduced the number of *E. Faecalis* colonies. The present study showed that CH had no considerable effect on *E. Faecalis* which confirmed previous studies about the resistance of *E. Faecali*s against CH [20]. In our study antimicrobial effects of TAP was higher than CHX gel in specimens from tubular depth of 200 and 400 µm. So it can be concluded that TAP penetrates the dentinal tubules better than CHX gel. 

The results of present study also indicated that TAP, including metronidazole, ciprofloxacin and minocycline, had an appropriate effect on *E. Faecalis* and this is in agreement with the study by Adl *et al.* [17] which was done in agar well diffusion test. Sotiriou *et al.* [22] indicated that antibacterial activity of NS particles and released silver ions against *Escherichia coli,* was comparable. Guangquan *et al*. [23] also showed that synthesized silver nanoparticles could efficiently inhibit bacteria and fungi. On the other hand, our results showed that NS gel could not eliminate *E. Faecalis* effectively. Probably this dissimilarity was caused by two main reasons; the different synthesis procedure of NS and the fact that the added gel may have inhibited nanoparticle ions from releasing. So we recommend further *in vivo* and *in vitro* studies on other microbial species to determine the antimicrobial effects of intracanal gel form medicaments.

## Conclusion

Under the limitations of this *in vitro* study, CHX gel and TAP can efficiently eliminate *E. Faecalis *as intracanal medicaments. On the other hand, CH gel, NS gel and normal saline are not effective against *E. Faecalis*.

## References

[1] Gondim JO, Avaca-Crusca JS, Valentini SR, Zanelli CF, Spolidorio DM, Giro E (2012). Effect of a calcium hydroxide/chlorhexidine paste as intracanal dressing in human primary teeth with necrotic pulp against Porphyromonas gingivalis and Enterococcus faecalis. International Journal of Paediatric Dentistry.

[2] Ballal NV, Yegneswaran PP, Mala K, Bhat KS (2011). In vitro antimicrobial activity of maleic acid and ethylenediaminetetraacetic acid on endodontic pathogens. Oral Surg Oral Med Oral Pathol Oral Radiol Endod.

[3] Mohammadi Z, Giardino L, Mombeinipour A (2012). Antibacterial substantivity of a new antibiotic-based endodontic irrigation solution. Aust Endod J.

[4] Anumula L, Kumar S, Kumar VS, Sekhar C, Krishna M, Pathapati RM, Venkata Sarath P, Vadaganadam Y, Manne RK, Mudlapudi S (2012). An Assessment of Antibacterial Activity of Four Endodontic Sealers on Enterococcus faecalis by a Direct Contact Test: An In Vitro Study. ISRN Dent.

[5] Razmi H, Aminsobhani M, Bolhari B, Shamshirgar F, Shahsavan S, Shamshiri AR (2013). Calcium Enriched Mixture and Mineral Trioxide Aggregate Activities against Enterococcus Faecalis in Presence of Dentin. Iran Endod J.

[6] Wang Z, Shen Y, Haapasalo M (2012). Effectiveness of endodontic disinfecting solutions against young and old Enterococcus faecalis biofilms in dentin canals. J Endod.

[7] Atila-Pektas B, Yurdakul P, Gulmez D, Gorduysus O (2013). Antimicrobial effects of root canal medicaments against Enterococcus faecalis and Streptococcus mutans. Int Endod J.

[8] Mohammadi Z, Shalavi S (2012). The effect of heat-killed Candida albicans and dentin powder on the antibacterial activity of chlorhexidine solution. Iran Endod J.

[9] Mohammadi Z, Shalavi S (2012). Is chlorhexidine an ideal vehicle for calcium hydroxide? A microbiologic review. Iran Endod J.

[10] Sinha N, Patil S, Dodwad PK, Patil AC, Singh B (2013). Evaluation of antimicrobial efficacy of calcium hydroxide paste, chlorhexidine gel, and a combination of both as intracanal medicament: An in vivo comparative study. J Conserv Dent.

[11] Sharifian MR, Shokouhinejad N, Aligholi M, Emaneini M, Katebi A, Assadian H (2008). In vitro comparison of the effectiveness of chlorhexidine and two calcium hydroxide formulations on enterococcus faecalis. Iran Endod J.

[12] Alt V, Bechert T, Steinrucke P, Wagener M, Seidel P, Dingeldein E, Domann E, Schnettler R (2004). An in vitro assessment of the antibacterial properties and cytotoxicity of nanoparticulate silver bone cement. Biomaterials.

[13] Shantiaee Y, Maziar F, Dianat O, Mahjour F (2011). Comparing microleakage in root canals obturated with nanosilver coated gutta-percha to standard gutta-percha by two different methods. Iran Endod J.

[14] Yasa I, Lkhagvajav N, Koizhaiganova M, Celik E, Sari O (2012). Assessment of antimicrobial activity of nanosized Ag doped TiO(2) colloids. World J Microbiol Biotechnol.

[15] Greulich C, Kittler S, Epple M, Muhr G, Koller M (2009). Studies on the biocompatibility and the interaction of silver nanoparticles with human mesenchymal stem cells (hMSCs). Langenbecks Arch Surg.

[16] Madhumathi K, Sudheesh Kumar PT, Abhilash S, Sreeja V, Tamura H, Manzoor K, Nair SV, Jayakumar R (2010). Development of novel chitin/nanosilver composite scaffolds for wound dressing applications. J Mater Sci Mater Med.

[17] Adl A, Shojaee NS, Motamedifar M (2012). A Comparison between the Antimicrobial Effects of Triple Antibiotic Paste and Calcium Hydroxide Against Entrococcus Faecalis. Iran Endod J.

[18] Taneja S, Kumari M, Parkash H (2010). Nonsurgical healing of large periradicular lesions using a triple antibiotic paste: A case series. Contemp Clin Dent.

[19] Grande NM, Plotino G, Falanga A, Pomponi M, Somma F (2006). Interaction between EDTA and sodium hypochlorite: a nuclear magnetic resonance analysis. J Endod.

[20] de Lucena JM, Decker EM, Walter C, Boeira LS, Lost C, Weiger R (2013). Antimicrobial effectiveness of intracanal medicaments on Enterococcus faecalis: chlorhexidine versus octenidine. Int Endod J.

[21] Dametto FR, Ferraz CC, Gomes BP, Zaia AA, Teixeira FB, de Souza-Filho FJ (2005). In vitro assessment of the immediate and prolonged antimicrobial action of chlorhexidine gel as an endodontic irrigant against Enterococcus faecalis. Oral Surg Oral Med Oral Pathol Oral Radiol Endod.

[22] Sotiriou GA, Pratsinis SE (2010). Antibacterial activity of nanosilver ions and particles. Environ Sci Technol.

[23] Rahimi S, Shahi S, Gholizadeh S, Shakouie S, Rikhtegaran S, Soroush Barhaghi MH, Ghojazadeh M, Froughreyhani M, Abdolrahimi M (2012). Bactericidal effects of Nd:YAG laser irradiation and sodium hypochlorite solution on Enterococcus faecalis biofilm. Photomed Laser Surg.

